# The Hydrological-Hydrochemical Factors that Control the Invasion of the Black Locust (*Robinia pseudoacacia* L.) in Succession in Areas with Opencast Mines

**DOI:** 10.3390/plants10010040

**Published:** 2020-12-25

**Authors:** Joanna Kidawa, Damian Chmura, Tadeusz Molenda

**Affiliations:** 1 Institute of Earth Sciences, Faculty of Natural Sciences, University of Silesia, 60 Będzińska St., 41-200 Sosnowiec, Poland; tedimolenda@interia.pl; 2 Institute of Environmental Protection and Engineering, Faculty of Materials, Civil and Environmental Engineering, University of Bielsko-Biala, 2 Willowa St., 43-309 Bielsko-Biała, Poland; dchmura@ath.bielsko.pl

**Keywords:** sand-gravel mine, primary succession, biological invasion, propagule pressure

## Abstract

Studies on opencast mines have indicated that the spontaneous colonization of excavations and sedimentation tanks by vegetation is determined not only by the substratum and the land relief, but also by the hydrological and hydrochemical relations in the exploitation hollow. Sometimes, biological invasions can also disturb the natural revegetation. *Robinia pseudoacacia* L. black locust is an invasive alien species that frequently colonizes sandy habitats. Thirty study plots were randomly established on four types of sites: (1) sandy sediments, extremely dry places located mainly on heaps of post-washer slime; (2) sandy sediments, dry areas that are periodically flooded and have pulp; (3) clay sediments, damp areas that are periodically submerged, and (4) the control, a forest with *R. pseudoacacia* in its neighborhood. A total of 94 species of vascular plants and seven species of mosses were found. The vegetation at the sites differs and the role of the black locust increases along the dryness gradient and developmental phase of vegetation. Older phases of succession resemble a forest in the surrounding area. It is a *R. pseudoacacia* species-poor monodominant stand that has been forming for around 30 years. A lack of trees and dense grasses favor the successful invasion of the black locust on man-made sandy habitats.

## 1. Introduction

Botanical studies that were conducted on opencast mines including sand mines revealed that spontaneous revegetation and forestry reclamation yielded congruent results: the species richness and the length of time required to achieve the stage of forest vegetation is similar. In addition, vegetation in abandoned sand mines can be more diverse in terms of species diversity [1]. On the other hand, local factors such as the bedrock [2,3,4,5,6] and hydrological conditions [7,8,9] can control the processes of spontaneous colonization. Depending on the geological substrate (e.g., gypsum, granite), the species composition of the flora is the result of the habitat requirements of the species that are colonizing excavations [10,11,12]. In sand pits where sand heaps and excavations, hydrological-hydrochemical conditions can differ. Xerothermic sand grasslands and the vegetation of therophyte swards tend to develop in dry places, i.e., on the top or slopes of heaps [13,14]. Usually, annual and stress tolerators prefer such extreme habitats [15], while reed beds and tall-sedge vegetation grow on the wetter sites that are situated at the bottom of excavations [16,17]. A similar phenomenon occurs in gravel opencast mines where sand is not the mineral being exploited, but is regarded waste that can be disposed of which form sand heaps. The aforementioned processes of natural revegetation can be disturbed by the appearance of invasive alien species. Usually, invasive species as well as ruderal plants can only occur during the younger successional stages and then disappear after a few years. However, under favorable conditions, some species such as *Robinia pseudoacacia* L. can develop and change the direction of succession and finally form a monodominant stand [18].

In this study we selected a site that is characterized by a wide diversity of morphological conditions (excavations, landfills, settling tanks) as well as water relations and hydrochemical characteristics. It is the opencast mine “Wójcice” where gravel and sand, which form bedrock of this area, are exploited. This mine, which is situated in southern Poland, has arable fields (corn plantations) and a forest in which *R. pseudoacacia* grows in close proximity. The territory of the mine has not yet been subjected to land reclamation because it is still active. Due to the legally sanctioned necessity to reclaiming degraded land in the future, the area of mine will be reclaimed. Three forms of management are being employed—forest reclamation, aquatic reclamation, and mining waste disposal [19]. Based on the conditions in the mine, it can be assumed that aquatic reclamation will probably be selected.

The aforementioned species, Black locust *Robinia pseudoacacia* L., is a legume tree that is native to North America and has been present in Europe since the beginning of 17th century and in Poland as a naturalized tree since mid-19th century. The species is considered to be invasive at least in nine European countries including, among others, Austria, Czechia, France, Germany, the Netherlands, Poland, and Switzerland [20,21,22]. It was deliberately introduced into Poland as an ornamental tree by gardeners, as a timber tree by foresters and as a way to improve the quality of the soil, namely, to enrich it with nitrogen. Currently, it is a species that is widespread in the country except for north-eastern part where it occurs less abundantly [23,24]. It frequently colonizes xerothermic meadow communities, dry forests, agricultural land, roadsides, as well as urban and industrial areas [20]. As a result of nitrogen fixation via symbiosis with the *Frankia* spp. and *Rhizobium* spp. bacteria in stands that are occupied by the Black locust the amount of nitrogen in the humus profile can be more than three-fold higher than it was originally [20,25,26]. Thus, it can change the properties of an ecosystem, which can lead to the displacement of oligotrophic and acidophilic species and the appearance of nitrophilic species [27,28]. It can also have an impact on the light conditions. In the stands in which this species dominates, the decrease in the size of the crowns is smaller and the cover of leaves is shorter than that of other deciduous trees [29]. This change in conditions promotes light-demanding species including herbaceous plants, especially grasses and geophytes, at the expense of the survival of the seedlings of shade-loving species, thereby strengthening and consolidating the effect of the conversion of a forest stand. Apart from plants, the species also has a negative influence on animals, e.g., birds. It was reported that it causes a decrease in the diversity of specialists to the benefit of the generalists [29]. *R. pseudoacacia* has invaded dry areas near disused gravel-sand mines. It is troublesome plant that can change the direction of spontaneous succession on these sites. Two final stages of vegetation development are also likely to form: shrubby grasslands or *Robinia* groves [18]. Taking into account that the species can also be encountered in alluvial habitats, we wondered whether it could thrive equally in both the dry and wet habitats that occur at the studied mine. Therefore, we studied the hydrological and hydrochemical properties of the water that could affect vegetation and observed the species composition of the vegetation along the environmental gradients in the vicinity of the mine.

The main objective of the research was to in order determine whether the natural succession at this mine is under pressure from *R. pseudoacacia*. The specific goals were to answer the following questions: How do the water affect the vegetation and *R. pseudoacacia* in the vicinity of the mine? Are there any differences in the contribution of *R. pseudoacacia* on the sites that differ in the amount of moisture and land relief? What is the approximate age at which the species forms a monodominant stand? What are the other environmental factors that control the development of the vegetation in the vicinity of the mine?

## 2. Results

### 2.1. General Hydrochemical Characteristics of the Water

**Figure 1:**
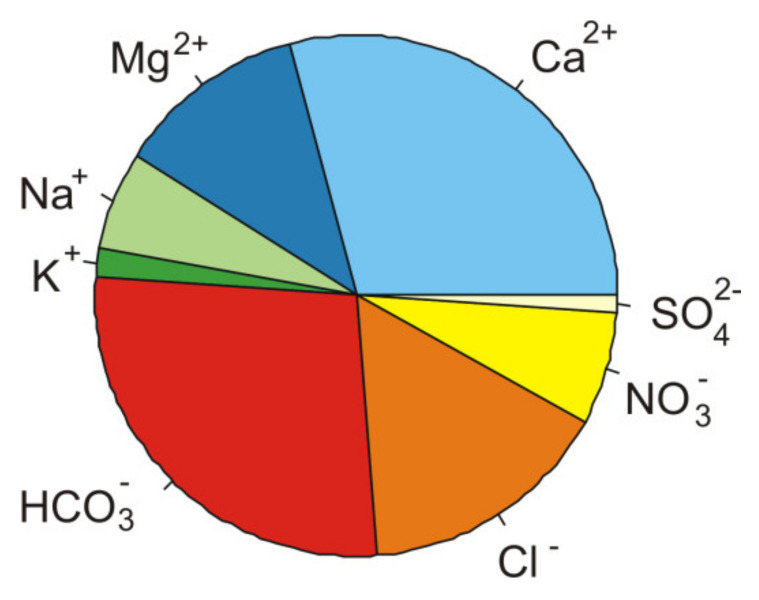
The chemical composition of the water in sedimentation tank is of the bicarbonate-calcium type.

The ionic composition of the water is dominated by bicarbonate anions (about 27%, 2.55 mval/L) and calcium cations (29%, 2.7 mval/L) (Figure 1). The water of the sedimentation tank is characterized by an average value of electrolytic conductivity of 360 μS/cm and neutral (pH~7) pH. A characteristic feature of the water is the trace amounts of biogenic substances—nitrogen and phosphorus. The average concentration of nitrates (NO_3_^−^) was only 1.5 mg/L and the concentration of phosphates (PO_4_^2−^) was below the limit to be quantified.

### 2.2. Characteristics of the Vegetation and Substratum on the Study Sites

On the study sites, a total of 101 taxa were found, including 94 vascular plant species, 7 moss species and lichens (*Cladonia* sp) (Supplementary Materials Table S1). Additionally, there was a cover of algae but the species were not identified. On the 30 research plots, from 3 to 20 (on average 8.9 species) were recorded per plot. Species of rush and reed beds (the *Phragmitetea* R. Tx. et Prsg 1942 class), sand swards (the *Koelerio glaucae-Corynephoretea canescentis* Klika in Klika et Novak 1941 class), meadow communities (the *Molinio-Arrhenatheretea* R.Tx. 1937 class) and scrub-forest were also found. On the study sites, which include SH—sand heaps, ST—a sandy bottom of the sedimentation tank, and WT—wetlands, there are several stages and processes that caused succession: colonization by algae and mosses on clay material in the first stage of succession around bird feces (study site WT) (Appendix A, Figure A2 and Figure A3) and colonization by grassland species on the desiccated parts of the settling tank (study site SH), while on the sandy material periodically flooded with pulp, algae and moss grow (study ST). In the second stage of succession, shrubs or biogroups of saplings (willows *Salix purpurea* L., *S. fragilis* L.—study site ST) occur and on study site WT, *S. purpurea* mainly occurs. According to the Indicator Species analysis (ISA), sand heaps are characterized by one indicatory species, *Picris hieracioides* L., whereas the grass *Cynodon dactylon* is the only species that is significantly affiliated to the sandy bottom of sedimentation tanks. The wetlands had five indicatory species and the highest number, nine, was growing in the adjacent forest, which was the control. The species *Oenothera biennis* L. and *Saponaria officinalis* L. had a high IndVal value in the two sandy habitats.

The grass *Calamagrostis epigejos* occurs significantly more often in the sand heaps and in the forest (Table 1). On the other hand, on the study site SH, on which the sand is stabilized by the roots of the grasses *Corynephorus canescens* and *Cynodon dactylon* L. dominated, Black locust (*Robinia pseudoacacia*) also occurs. The final stage of succession is the formation of black locust thickets or a black locust forest (study site SH), willow scrubs (study site ST), and a mosaic of rushes and willow scrubs (study site WT).

**Figure 2:**
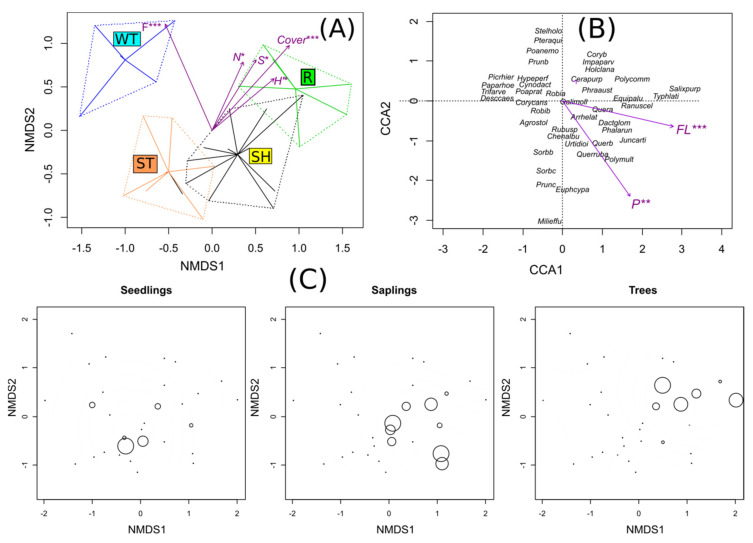
Nonmetrical Multidimensional Scaling (NMDS) and Canonical Correspondence Analysis (CCA). NMDS centroids (**A**), biplot of the study plots and environmental factors according to the CCA (γ1 = 0.5954 γ2 = 0.5443 and for which the cumulative explained proportion was 0.3350 and 0.6413 for the two axes, respectively) (**B**) and ordination of the *Robinia pseudoacacia* in relation to the layer and cover (**C**). Explanations: Cover—total cover of plants, S—species richness, H’—Shannon-Wiener index, N—nitrogen, FL—floatable parts fraction, *—*p* < 0.05; **—*p* < 0.01, ***—*p* < 0.001. 1 R—reference site, adjacent forest, SH—sand heaps, ST—sandy bottom of the sedimentation tank, WT—wetlands.

A Nonmetrical Multidimensional Scaling (NMDS) analysis showed a large variation in the species composition of vegetation among the study sites. The vegetation of the study sites differed significantly from each other according to a centroid analysis using passive vector matching (r^2^ = 0.74, *p* = 0.001). The total cover of plants, species richness, and the value of Shannon-Wiener significantly explained gradient of the vegetational changes from a wetland to forest communities (*p* = 0.001, *p* = 0.013, *p* = 0.011), respectively. The Ellenberg indicator values (EIV) indicators for moisture and nitrogen were also significant (*p* = 0.001, *p* = 0.012), respectively. All of these variables are associated with the first axis of NMDS (Figure 2A).

According to the Canonical Correspondence Analysis (CCA), the only significant habitat factors are the content of floatable parts (*p* = 0.001) and the content of phosphorus (*p* = 0.001). Higher values of these factors are associated with the wetland plots on clay material and to some degree with the forest plots (Figure 2B). On these sites, there were differences in the frequency of mature trees and saplings (G = 9.858, *p* = 0.01981, and G = 11.366, *p* = 0.009904), respectively, whereas the differences in the frequency of the seedlings of *R. pseudoacacia* among the habitats were not significant (G = 2.7291, *p* = 0.4353). There were no significant differences in the cover abundance. Only a few individuals of Black locust were found in the WT and the ST habitats (Figure 2C).

**Figure 3:**
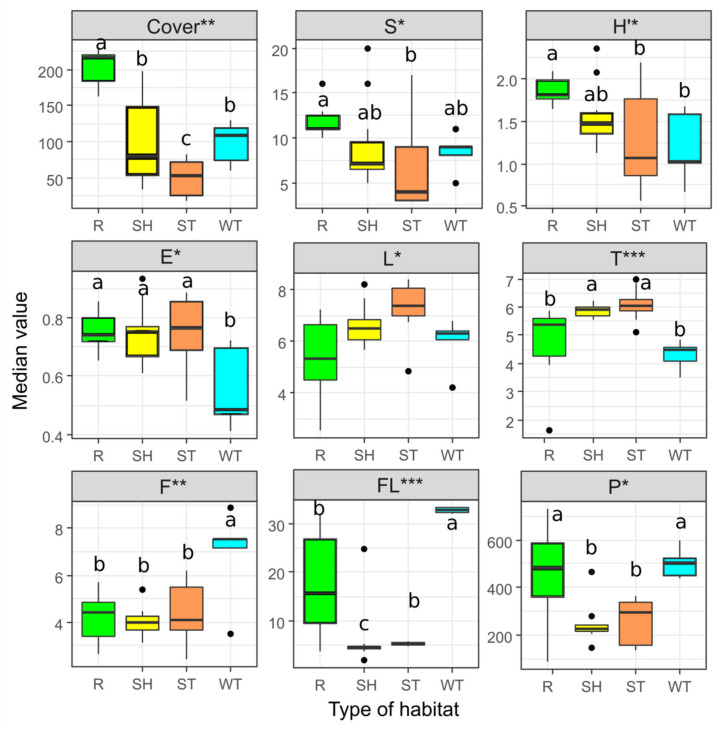
Comparison of the total cover of plants (COVER), species richness (S), Shannon-Wiener index (H’) and evenness index (E); Ellenberg indicators for light (L), temperature (T), and moisture (F); soil characteristics for phosphorus (P), and floatable parts (FL), among study sites. R—reference site, adjacent forest, SH—sand heaps, ST—sandy bottom of sedimentation tank, WT—wetlands. Significance of the differences according to the Fisher-Pitman test *—*p* < 0.05; **—*p* < 0.01, ***—*p* < 0.001. The same lowercase letters indicate no significant difference.

There were significant differences in the total plant cover. The highest mean values were found for the study plots in the adjacent forest and the lowest were found on the sandy sediments that are flooded with pulp. The highest value of the Shannon-Wiener index was found for the forests and heaps of sand, and the lowest for the wetlands and the sandy bottom of the sedimentation tank. The highest EIV values for light and temperature were observed for the sandy bottom of the sedimentation tank followed by the sand heaps. The EIV for moisture was highest in the wetlands and they also were the characterized by the highest content of phosphorus and floating parts (Figure 3). Differences in other environmental variables (pH, total nitrogen) were not statistically significant.

## 3. Discussion

### 3.1. Hydrochemical Assesment of Water and Pulp

The detected double-ion domination of bicarbonate ions and calcium is a common hydrochemical type of water that occurs in nature [30]. The values of electrolytic conductivity and pH are comparable to those found in most anthropogenic water bodies [31]. The trace amounts of nutrients in the water are another factor that limits colonization. The lack of nutrients in the water is a consequence of the closed water cycle in the sedimentation tank. The water flows through reeds that are located in the sedimentation tank, which removes nutrients from the water. In this case, the sedimentation tank acts as a “hydrophytic treatment plant”. The high efficiency of the settling tank also purified the suspended matter. In the pulp discharge zone, the water turbidity exceeded 100 NTU and in the re-entry zone was about 50 NTU. The periodic flooding with water in the settling tank affects the development of the vegetation on study site WT (wetland) and sporadically on study site ST (sedimentation tanks).

### 3.2. Type of Succession of the Vegetation in the Vicinity of the Open-cast Mine

**Figure 4:**
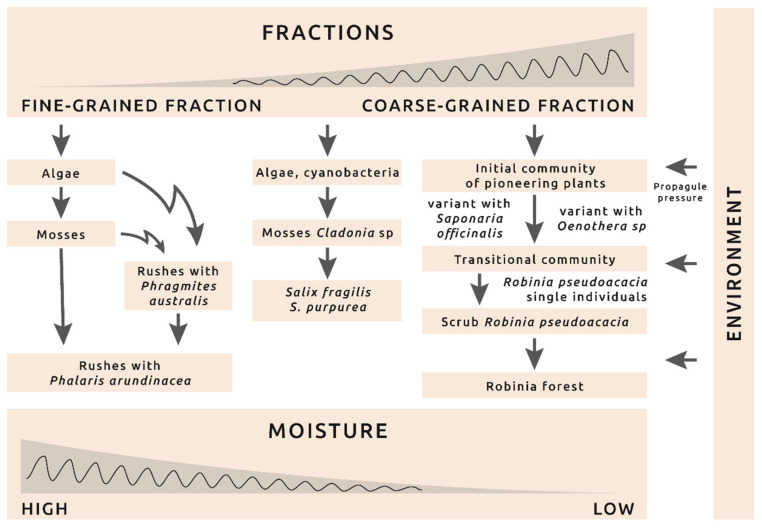
Model of succession: directions and phases of vegetation development in the vicinity of the “Wójcice” mine.

The development of vegetation in the vicinity of the “Wójcice” mine has the character of primary succession because it begins on bare ground without a plant cover. Several different directions of the succession and stages of vegetation in the vicinity of the mine can be distinguished (Figure 4). These are facilitation processes that mainly result in the modification of the habitat by pioneer species [32]. The direction of succession primarily depends on the hydration and type of substrate material, which is well illustrated by comparing not only the vegetational gradient (Figure 2A) but also the effect on the species composition and differences in the fraction of floatable parts (Figure 2B). The floatable parts correlated with the first CCA axis. While we did not determine moisture, its indirect proxy is the fraction of floatable parts. The higher the content of the fractionable fraction, the more the substratum absorbs water. Therefore, it is not surprising that mainly rush vegetation develops (reed and canary beds) on the clay material. On the other hand, there are sandy places that are overgrown with plants that are typical for sandy swards of the *Koelerio glaucae-Corynephoretea canescentis* class that have a large contribution of *Corynephorus canescens*. They develop best on sunny slopes where the soil temperature is higher. The high content of sands and gravels means that the substrate dries quickly even after heavy rains [33]. In the area of the sandy sediments that are periodically flooded with pulp, there are places with a large fraction of floating parts, which can be seen as the outlier on Figure 3. At the bottom of the sedimentation tank, which carry a lot of clay materials, periodic rivers occur. The sandy grasslands in the vicinity of the mine are quite species poor. The plots had an average of 9.1 species and the average value of the Shannon-Wiener index was 1.5. In the review paper by Sienkiewicz-Paderewska [34], the examined communities of the *Koelerio glaucae-Corynephoretea canescentis* class were characterized by an average number of species in the phytosociological relevé between 8.0 and 18.6 and the value of the Shannon-Wiener index was 1.5 and 3.5. The observed patches of grasslands in the vicinity of the mine are close to the lower values in that paper. In the patches of the grasslands of the vicinity of the sandy mine, there are several variants of the initial stages with *Corynephorus canescens*, *Saponaria officinalis*, *Oenothera biennis*, and *Senecio viscosus*. There are also intermediate patches that contain a mosaic of the above-mentioned species. The presence of the *Cynodon dactylon* is noteworthy because it is an ephemerophyte, a species that was introduced into Poland in 1825, which probably originated in Africa. Although it is still not naturalized, it has over 30 stands in Poland mainly in sandy areas [35].

The wetland habitat where study site WT was established differs in both moisture and material—mainly clays. In addition, animal activities play an important role in a succession. It seems that the processes of habitat modification and the facilitation of succession are initiated by bird excrement and algae (enrichment of the habitat in nutrients) and that the ground is stabilized by willow roots and their leaves falling. At the shoreline of the tank, bird tracks (mainly ducks) and their excrement and algae colonies form around them. The impact of bird feces on the circulation of nutrients and the formation of ecosystems has been described in many regions of the world and environments, e.g., in forests [36], in water reservoirs [37], or even in Antarctica around penguin colonies [38]. Bird feces are primarily a source of phosphorus. This is confirmed by our research, where the highest reported values of phosphorus are in the area of wetlands (Figure 3). They are also high in the soil in the forest in the vicinity of the mine. The rush communities that occur in the vicinity of the mine are characterized by a small number of species in the relevé from 5 to 11 with an average of 8.4 and the Shannon-Wiener index is 1.19. This is typical for the rush communities here, which are mainly built by the reed *Phragmites australis* or the reed canary grass *Phalaris arundinacea* [39]. They are mainly monospecific patches with a large total coverage of plants as was the case in this study (Figure 3).

The places that periodically flooded with pulp, which are outside the coastal zone of the water reservoir is where the patches of communities built by willows formed: the crack willow *Salix fragilis*, the purple willow *S. purpurea*, and the almond willow *S. trandria*. They are mainly shrub communities and there are only a few species per plot where an important phenomenon occurs, namely leaf fall. There is an enormous body of literature about the role of leaf decomposition and its impact on the soil properties specifically enriching the soil with nutrients and circulating matter [40]. Compared to the plots on the sand heaps, those with willow are characterized by higher values of nitrogen and phosphorus (Figure 3).

### 3.3. Invasion of Robinia Pseudoacacia

In the later stages of succession in the vicinity of the mine the Black locust *Robinia pseudoacacia* occurs primarily on the sands (study sites 1 and 2) (Figure 2B). In the vicinity of the mine, Black locust individuals in almost all stages of succession occur. Various herbaceous species grow under the canopy of trees, first forming biogroups, then thickets, and finally a compact Black locust forest. The estimated time for this type of forest to develop is about 25 years (based on the observation of the mine personnel). This is a shorter time compared to other results [41] where succession proceeded toward stabilized, (semi-) natural vegetation with an *R. pseudoacacia* contribution within 40 years. The thickets and forest with *R. pseudoacacia* are characterized by a lower species richness (maximum 20 species per plot) and low values of Shannon-Wiener index, which is congruent with the general trend. In other habitats, even those with natural forest conditions, the *Robinia* forest were species poor [42]. In our study, the largest individuals in this phase of the Black locust forest on sandy habitats were about 8 m high and had ca 5 to 13 cm of diameter at breast height (DBH), whereas the largest stems in adjacent forest had a DBH that was no larger than 16 cm. Thus, all of them can be classified as early-successional trees and shrubs of Black locust [43]. The research results show that the older stages of succession, especially on the sand heaps, are similar to the forest in the area in terms of its structure and species composition (Figure 3). Based on the species composition, it can be concluded that the adjacent forest resembles the human-cultivated forest *Chelidonio-Robinietum* Jurko 1963 s.l. [43,44]). Taking into account the relatively small distance from the adjacent forest to the territory of the mine including the sandy habitats, the transport of seeds by the wind could be efficient enough to disperse this plant. It was reported that seeds can be transported by wind on the ground up to 67 m from a single mother plant [43]. The nearby forest with *R. pseudoacacia* is a probable source of the propagules of black locust in the habitats in the vicinity of the sand-gravel mine. The presence of water directly affected the performance of *R. pseudoacacia*. It was not found in the distinguished study plots in the areas that flooded. This contradicts the findings that they can occupy alluvial sites and previously flooded area [20]; however, these authors also state that the species does not occur in wetlands or the parts of alluvial forests that have frequent and long-term waterlogging and in which the soils are compact. In the present study, *R. pseudoacacia* was not found anywhere in the wetlands and in areas that got flooded, there are only a few individuals. The hydrochemical properties of water do not matter at all, especially because they are typical for the majority of surface water in Poland.

A summary of the directions of succession and individual phases of the development of vegetation is presented in the diagram in Figure 4. The processes of colonization and succession in this study resemble the processes that was described in the Błędowska Desert by Rahmonov [45]. Similar to that study, this is a primary succession on sand where algae, mosses, and vascular plants that are typical for dry, xerothermic habitats play an important role. It is also typical that the soil development is slower than the development of the plant cover. It is noteworthy that the changes are progressive in terms of the increase in the number of species and coverage. This pattern is consistent with most similar studies [18,41], but is contrary to the results of Kompała-Bąba and Bąba [46] where the number of species decreased and the role of dominants increased in a sand pit.

## 4. Materials and Methods

### 4.1. Description of the Study Area and Study Design

**Figure 5:**
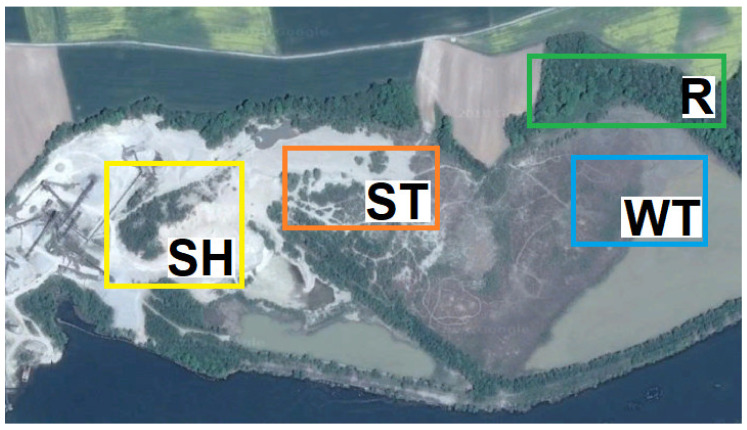
The spatial distribution of the study plots in the vicinity of the “Wójcice” mine. Explanations: study site SH—zone of sand heaps, extreme dry conditions, advanced succession—11 study plots, site ST—bottom of the sedimentation tank, zone of sand deposits, initial and mid-advanced succession (maybe periodically flooded by pulp)—8 study plots, site WT—zone of clay deposits, moist areas, periodically flooded, various forms of succession—5 study plots and site R—control, situated in the adjacent forest—6 study plots.

**Figure A1:**
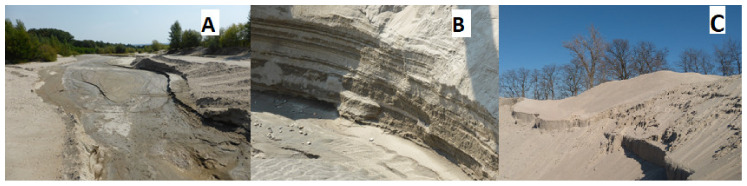
(**A**) Place of the pulp dump into settlement tank which creates the periodic and braided river. (**B**) Characteristic laminated sand sediments deposited on the settling tank. (**C**) Sand heaps (slurry).

**Figure A2:**
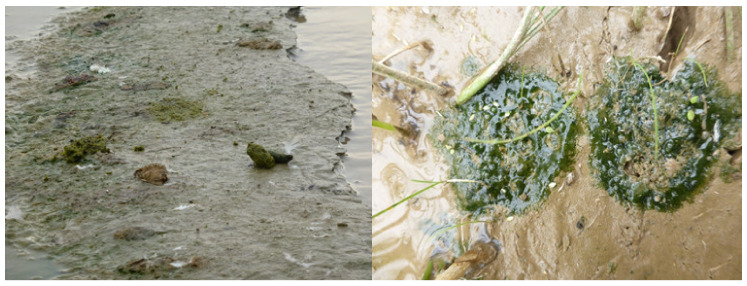
Algae colonies around the bird feces on the clay material, first stage of succession and example of habitat modification.

**Figure A3:**
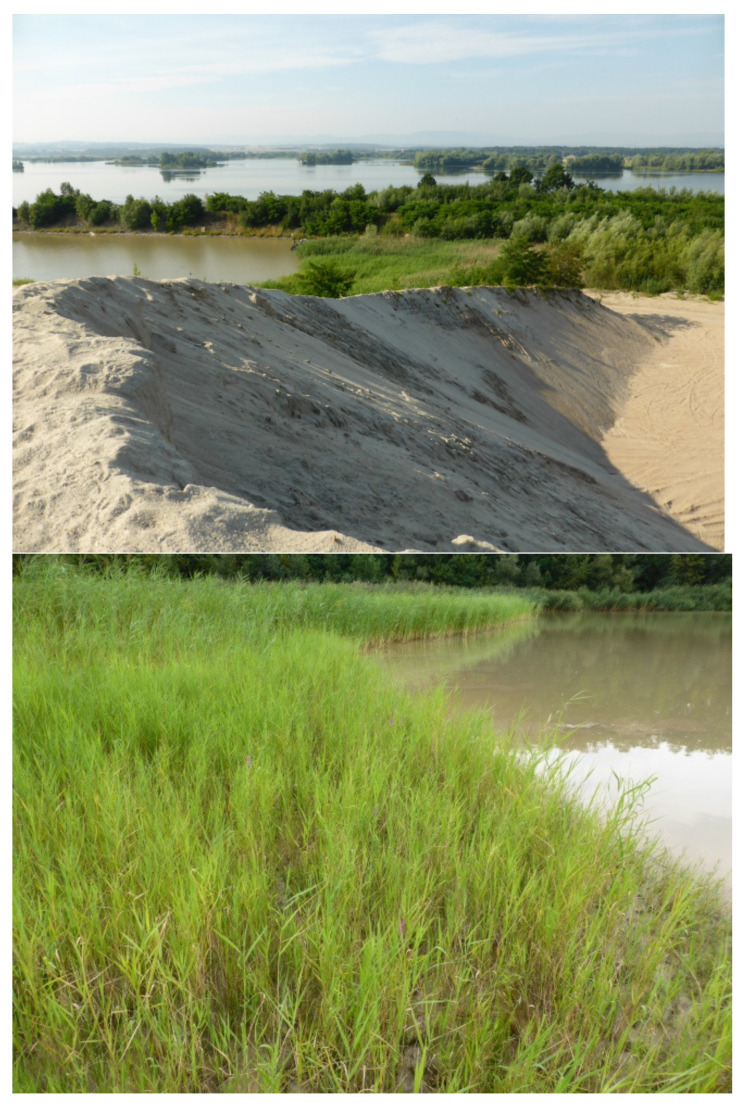
Two extreme habitats: sand heaps and wetlands on the territory of the open cast mine.

The study area (the Wójcice mine) is the territory of an open cast mine. The mineral aggregate deposits are mined using the underwater method, which is conducted in the dam reservoir zone of the “Nysa Lake”. The output is rinsed and screened (sorted) in order to remove the smallest fractions (mainly sand and clay). While the water for rinsing is taken from “Nysa Lake”, it is circulated in a closed cycle. After passing through the sedimentation tank, it is used in material sorting processes once again. The lake water is only used to compensate for any technological losses. Of the mineral aggregates that are mined, only the gravel is sold. As a result, the fractions of highly hydrated pulp that are separated are hydraulically transported to the sedimentation tank where it undergoes sedimentation and dehydration (Figure A1A, Appendix A). The pulp is a by-product of the mine, which cannot be sold due to its specific properties. In the sedimentation tank, thickest fractions are separated in the first part of the sedimentation (sands with a small admixture of gravels) after which the fine-grained dust and clay fraction is separated (mainly in the water zone of the settling tank). The sludge that is deposited in the settling tank is characterized by characteristic lamination, which is a consequence of cyclic pulp inflows (Figure A1B). Recently, however, the sandy sediments that is deposited in the settling tank has also been exploited. This is called the exploitation of the anthropogenic deposit, which are primarily heaps of sand that are unstabilized and heavily desiccated (Figure A1C, Appendix A). The analysis of the vegetation mapping, type of substratum, degree of moisture and age of the sedimentation tank enabled four research study sites to be distinguished (Figure 5) and within them 30 study plots (Table 2). These were 1—sand heaps (SH), 2—sandy bottom of sedimentation tank (ST) 3—wetlands (WT), and 4—reference site (R, adjacent forest). The reference site was a forest with a high contribution of *R. pseudoacacia* in its tree layer. Based on the species composition, the forest can be classified as a degenerated oak-hornbeam forest *Tilio-Carpinetum*. Taking into account the internal diversity of the succession stages and the surface area of the distinguished habitats, a total of 30 study plots of 5 m × 5 m were established. During the vegetation season from the end of March to the end of August, soil and botanical studies were carried out and at the site of the discharge of the pulp onto the surface of the settling tank, hydrochemical studies of water were performed.

### 4.2. Methods of the Hydrological Studies

The hydrology was mapped according to the guidelines by Gutry-Korycka, Werner-Więckowska [47]. During the research, ten water samples with the pulp that were floating to the surface of the settling tank were collected. The basic physical and chemical properties of water (temperature, pH, oxygen saturation) and redox potential were measured directly in the field using a YSI EDS 6600 multi-parameter probe (US production). Prior to each test, the probe was calibrated using the standard solutions. During the fieldwork, water samples were also collected for the chemical analyses. The samples for the laboratory analyses were placed into 0.5 L polyethylene bottles. The water samples were transported to the laboratory at +4 °C. They were filtered on a 0.45 μm filter (Millipore). Laboratory analyses included determining the main cations and anions in the water: Ca^2+^, Mg^2+^, Na^+^, K^+^, NH_4_^+^, HCO_3_^−^, SO_4_^2−^, Cl^−^, NO_3_^−^ and PO_4_^2−^. The analyses were performed using a Metrohm 850 Professional IC ion chromatograph. The hydrochemical type of the water was determined based on the classification of Szczukariew-Prikłoński [48].

### 4.3. Methods of the Soil Analyses

Composite soil samples were taken from the study plots and the physical-chemical properties as well as the granulometric composition of soil was determined: pH (in an aqueous solution and in a 1N KCl solution), N_T_—total nitrogen according to PN-ISO 11261, P—available phosphorus according to PN-R-04023, and FL—fraction of floatable parts using the Prószyński’ aerometric method PN-R-04033 [49].

### 4.4. Methods of the Botanical Studies

Within the study plots, a floristic inventory was taken (vascular plants and bryophytes) and a modified Braun-Blanquet approach was performed [50]. In total, 30 phytosociological relevés were taken at all of the distinguished study sites. The cover abundance of the recorded plants was visually estimated in % (0.5, 1, 2, 5, 10, 20 ... 100) and the total cover using the Canopy Cover Free 1.03 application for mobile devices with the Android system and canopyscope [51] with our own modifications [52] were used to assess the tree canopy. Based on the occurrence and cover abundance of the species, the Ellenberg indicator values (EIV) were calculated [53]. The cover-weighted EIV were calculated for light (L), temperature (T), moisture (F), and soil reaction (R). The EIV for nitrogen (N) was not considered because the soil samples were analyzed for N_T_ and the continentality K was not applicable in this study. The nomenclature for the species follows the Euro+MedPlantBase [54], whereas the syntaxonomical nomenclature follows the Guide for Plant Communities in Poland [55].

### 4.5. Statistical Analysis

The statistical analyses and visualizations were performed in the R software [56]. The Indicator Species Analysis (ISA) using indicator value, i.e., IndVal—the indicator value that indicates the magnitude of species affinity and the p-value of the statistical significance were also calculated (package *indicspecies*). Based on the collected vegetation data, the biodiversity indices were calculated: the number of species (S), the Shannon-Wiener diversity index (H’), and the species evenness (E), as well as the sum of the species cover (COVER). Next, Nonmetric Multidimensional Scaling (NMDS) was conducted to examine the variability direction of the analyzed vegetation. For the NMDS, the data was log-transformed. In order to examine the differences in the species composition of vegetation among the four study sites, the centroids representing the vegetation of a given study site was analyzed using the vector fitting method. The same test (999 permutations) was used to fit the biodiversity indices to the ordination result as passive vectors, which were plotted as an NMDS biplot. In order to investigate the influence of the habitat factors pH, total nitrogen content, phosphorus and percentage fraction of the floating parts of the substratum (fraction < 0.05 mm) on the species composition, a canonical correspondence analysis (CCA) was performed, and the significance of the habitat factors was also assessed using the permutation test (999 iterations). The biodiversity indices and ordination were analyzed using the *vegan* package. In turn, the significance of the differences in the biodiversity indices and habitat factors was analyzed using a non-parametric equivalent of the analysis of variance ANOVA, i.e., Asymptotic K-Sample Fisher-Pitman Permutation Test [57] followed by a post-hoc Conover test. The significance of the differences in the frequency of *R. pseudoacacia* was tested by G-test using the *DescTools* package. The significance was assumed at a level of *p* < 0.05.

## 5. Conclusions

The various phenomena of the colonization and vegetation succession that occur in the vicinity of the mine were determined by three main factors: the type of substrate, especially the type of the fraction (sands and clays), hydration (overdried areas vs. areas that are flooded with water), and the environment (degenerated oak-hornbeam forest). The development of the plant cover was faster than the soil cover formation, which is shown by the example of succession on sands. On the overdried sites, the older stages of succession resemble the forest with *R. pseudoacacia* in the vicinity of the mine. The occurrence of this species in the study area was possible due to the propagule pressure (fruits, seeds) of *R. pseudoacacia* from the adjacent forest, which was confirmed by other studies. *R. pseudoacacia* is only able to invade dry sites, and on wet sites, its spread and abundance is strongly constrained. On flat sites that are flooded with water, only willow (*Salix* spp) can thrive. Thus, differences in hydrology causes a niche shift of woody species.

## Figures and Tables

**Table 1:** The estimates of the indicator values of the species in a specific type of vegetation. *—*p* < 0.05; **—*p* < 0.01, ***—*p* < 0.001.

Habitat	IndVal	Habitat	IndVal
Sand heaps (1)		Control (4)	
*Picris hieracioides* L.	0.603 *	*Rubus sp*	0.683 *
Sandy bottom of the sedimentation tank (2)		*Quercus robur* a L.	0.665 *
*Cynodon dactylon* L.	0.612 *	*Arrhenatherum elatius* (L.) P.Beauv. ex J.Presl and C.Presl	0.578 *
Wetlands (3)		*Convallaria majalis* L.	0.578 *
*Equisetum palustre* L.	0.894 ***	*Corylus avellana* b L.	0.578 *
*Epilobium cilatum* Raf	0.756 **	*Euphorbia cyparissias* L.	0.577 *
*Phragmites australis* (Cav.) Trin. ex Steud.	0.731 *	*Impatiens parviflora* DC.	0.577 *
*Carex nigra* (L.) Reichard	0.632 *	*Polygonatum multiflorum* (L.) All.	0.577 *
*Lythrum salicaria* L.	0.632 *	*Quercus rubra* a L.	0.577 *
1 + 2		1 + 4	
*Oenothera biennis* L.	0.761 *	*Calamagrostis epigejos* (L.) Roth	0.686 *
*Saponaria officinalis* L.	0.753 *		

**Table 2:** The characteristics of the study sites.

Habitat	Moisture	Grain Fraction	Species
Sand heaps (SH), n = 11	low	coarse	*Saponaria officinalis, Senecio viscosus, Corynephorus canescens, Oenothera sp., R. pseudoacacia*
Sandy bottom of the sedimentation tank (ST), n = 8	high	coarse to fine	*Salix fragilis, S. purpurea, Corynephorus cansecens, Oenothera sp*
Wetlands (WT), n = 5	high	fine	*Phragmites australis, Equisetum palustre,*
Control (R), n = 6	medium	medium	*R. pseudoacacia, Quercus robur, Rubus* sp.

## Data Availability

Data available on request from the authors. The data that support the findings of this study are available from the corresponding author upon reasonable request.

## Supplementary Materials

The following are available online at https://www.mdpi.com/2223-7747/10/1/40/s1, Table S1: Species data with mean and SD percent cover and type of occupied habitat.
